# A cross-sectional online survey evaluating how anaesthesiologists view cardiovascular magnetic resonance imaging reports as a tool for perioperative risk evaluation

**DOI:** 10.1016/j.bjao.2026.100649

**Published:** 2026-07-31

**Authors:** Kady Fischer, Raul Harms, Josef Nunzio Fini, Thilo Schweizer, Dominik P. Guensch

**Affiliations:** Department of Anaesthesiology and Pain Medicine, Inselspital, Bern University Hospital, University of Bern, Bern, Switzerland

**Keywords:** cardiovascular magnetic resonance, cardiovascular risk factors, clinical practice, perioperative risk evaluation, survey

## Abstract

**Background:**

Cardiovascular complications are a leading cause of perioperative adverse outcomes, particularly in patients with pre-existing cardiovascular disease. Anaesthesiologists routinely assess cardiovascular risk preoperatively, yet the use of cardiovascular magnetic resonance (CMR) findings in this context remains unclear. This study evaluated how anaesthesiologists perceive and use preexisting CMR findings for perioperative risk evaluation.

**Methods:**

We conducted an online survey for anaesthesiologists. The primary endpoint was incorporation of CMR findings into perioperative decision-making, with secondary analyses examining factors associated with CMR use and understanding of CMR parameters.

**Results:**

A total of 455 anaesthesiologists from 32 countries completed the survey. Most were board certified (84%), and 56% indicated cardiovascular or thoracic anaesthesiology (CVA) as a clinical activity. Overall, 68% reported incorporating findings from CMR reports for perioperative risk assessment at least sometimes, with no difference in use between respondents specialising in CVA and those who do not (69% *vs* 67%; odds ratio 1.17 [95% confidence interval 0.84–1.63]; *P*=0.36). Although left ventricular anatomy and function were widely incorporated (72%), understanding of advanced tissue characterisation markers was limited, particularly among non-CVA anaesthesiologists. Notably, 74% agreed that CMR holds potential for perioperative risk evaluation, and 54% expressed interest in its future integration.

**Conclusions:**

Among respondents to this voluntary survey, CMR findings were frequently reported as being incorporated into perioperative risk evaluation, including those without CVA specialisation. However, use remains focused on conventional functional metrics, with limited knowledge of advanced CMR markers. Further research linking CMR parameters to perioperative outcomes, alongside targeted education initiatives, is needed to enable evidence-based implementation in anaesthetic practice.

**Editor's key points**

• Cardiovascular magnetic resonance (CMR) is increasingly available in routine clinical practice, yet its use by anaesthesiologists during perioperative risk assessment has not been well characterised.
• Among 455 anaesthesiologists from 32 countries, more than two-thirds reported incorporating CMR findings into perioperative cardiovascular risk assessment when reports were available.
• Use of CMR findings was associated with local imaging infrastructure and familiarity with cardiovascular imaging modalities.
• Anaesthesiologists reported a greater understanding of conventional measures of cardiac anatomy and function than of advanced myocardial tissue characterisation techniques.
• The survey highlights growing interest in perioperative applications of CMR and identifies a need for evidence linking CMR-derived markers to perioperative outcomes.

Perioperative cardiovascular complications remain one of the leading causes of morbidity and mortality in surgical patients.^1^^,^^2^ Anaesthesiologists are responsible for the perioperative management of surgical patients and face an increasing challenge of optimising perioperative care for patients with complex cardiovascular profiles, who are at elevated risk of haemodynamic stress and adverse cardiac events during surgery.^2^ Anaesthesiologists mitigate this risk by performing preoperative cardiovascular risk stratification; however, this task has become increasingly challenging in the context of an ageing population and the increasing prevalence of cardiovascular disease.^3^ This trend is evident not only in cardiac surgery but also in noncardiac surgery, where patients may also present with multiple cardiovascular risk factors.^4^^,^^5^ This underscores the importance of effective cardiovascular risk assessment and management across all surgical settings.^1^^,^^2^

Preoperative cardiovascular risk evaluation aims to estimate the likelihood of perioperative adverse events to guide decisions regarding surgical suitability, additional testing, and perioperative monitoring. Risk evaluations integrate clinical history, physical examination, assessment of functional capacity, biomarkers, and established risk scores such as the ASA physical status classification and the Revised Cardiac Risk Index. Although these tools contribute valuable information, no single marker consistently delivers high predictive accuracy across all patient populations and surgical contexts.^6^^,^^7^ Cardiovascular imaging therefore represents an important adjunct. Echocardiography is widely accessible; however, it does not assess myocardial tissue characteristics. In contrast, cardiovascular magnetic resonance (CMR) imaging offers comprehensive multiparametric evaluation, including assessment of ventricular volumes and function, myocardial perfusion and inducible ischaemia, detection of acute myocardial injury via oedema imaging, and identification of chronic myocardial damage via scar characterisation.^8^, ^9^, ^10^, ^11^, ^12^, ^13^

Although anaesthesiologists do not typically request CMR for preoperative assessments, they are increasingly likely to encounter CMR findings during routine review of patients' medical records, driven by the increasing number of diagnostic CMR examinations performed^14^ and improved access to reports from externally performed examinations through electronic medical record exchange across institutions. Despite this, little is known about how anaesthesiologists interpret and apply CMR reports in perioperative decision making, particularly in the absence of dedicated evidence or guidelines. In late 2022, the European Society of Cardiology (ESC), endorsed by the European Society of Anaesthesiology and Intensive Care, released guidelines on the cardiovascular assessment and management of patients undergoing noncardiac surgery, acknowledging the role of stress imaging, including CMR, in select patients.^15^

Against this background, the utilisation, understanding, and perceived value of CMR findings among anaesthesiologists remain poorly characterised. Therefore, this study aimed to assess how anaesthesiologists currently interpret and incorporate CMR findings into perioperative cardiovascular risk assessment and their perception of the potential future role of CMR in anaesthetic practice. By addressing this knowledge gap, the study also sought to inform future research and support closer interdisciplinary collaboration between anaesthesiology and cardiovascular imaging specialists.

## Methods

### Study design and setting

This anonymous voluntary survey was designed to be completed online in less than 10 min using the REDCap® (Research Electronic Data Capture) survey form.^16^ It was an open survey, provided only in English and consisted of up to 35 multiple-choice questions, some of which allowed multiple responses, along with a few adaptive questions that were displayed based on answers related to other questions. Most questions followed a Likert-type scale. Up to nine questionnaire items were displayed per page, with nine pages in total. Questions were not randomised, and respondents could skip questions or navigate back to previous pages, allowing for review or changes to answers. Completing the questions was not required to submit the survey. Once the survey was closed, it could no longer be accessed. No identifying information was collected from participants, and device information was not collected to identify potential duplicate entries. No time stamps were assessed to investigate atypical response times. All survey questions are presented in the Supplementary Tables.

### Participants and eligibility criteria

Eligible participants were clinicians who had actively worked at least 50% in anaesthesia care during the previous 2 years.

### Survey development and validation

The survey was developed by a panel of scientists and junior and senior anaesthesiologists, with input from the University of Bern Department of Clinical Research. Two validation rounds were performed in which anaesthesiologists from various specialities of the local department were asked to complete the survey and provide feedback on the content and clarity of the questionnaire, after which the panel adjusted the survey.

### Survey distribution and recruitment

The survey was endorsed and distributed by the Swiss Society for Anaesthesiology and Perioperative Medicine and the European Association of Cardiothoracic Anaesthesiology and Intensive Care (EACTAIC). For both societies, at least three e-mails about the survey were sent to their respective member lists, and for the EACTAIC, a link to the survey was posted on its website. The survey was further distributed on the International Anesthesia Research Society Online Communities Platform, by the Rural Society of Physicians in Canada; the French Society of Anaesthesia, Critical Care and Perioperative Medicine; and institutional departments. The electronic link was accessible from May 2024 to March 2025. The survey invitation is presented in the Supplementary Methods.

### Outcomes

**Fig 1:**
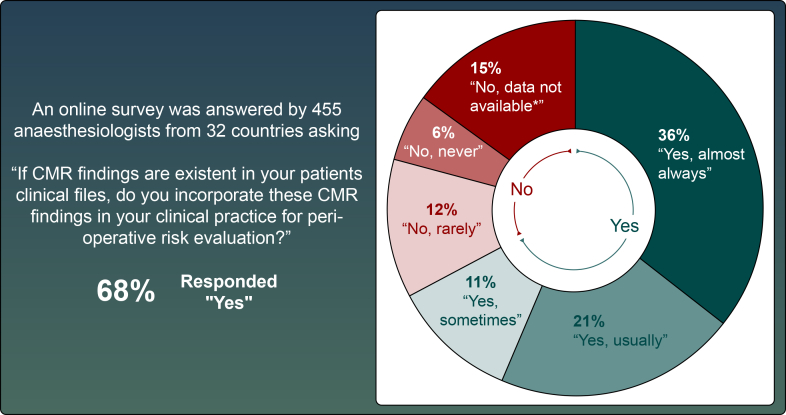
Overview illustrating the use of CMR reports in perioperative risk evaluation by survey respondents. An online survey was voluntarily answered by anaesthesiologists from 32 countries, asking multiple questions about integration of CMR into perioperative risk evaluation, from which 68% of responders answered ‘yes, almost always’, ‘yes, usually’ or ‘yes, sometimes’. Analysis was not adjusted for respondent characteristics. ∗Full text for response is ‘To the best of my knowledge data is not available in my patient files’. CMR, cardiovascular magnetic resonance imaging.

The primary outcome was overall responses to the following question: ‘If CMR findings are existent in your patients’ clinical files, do you as an anaesthesiologist incorporate these CMR findings in your clinical practice for perioperative risk evaluation?’ There were six possible responses (Fig 1), and a positive response was defined when anaesthesiologists answered ‘yes, always’, ‘yes, usually’, or ‘yes, sometimes’. For statistical analysis, all responses were considered as independent entities. The term ‘incorporation’ was not further specified and was left to the respondent's interpretation.

Anaesthesiologists were also asked to provide information regarding their individual expertise and institutional infrastructure. These variables were used to assess secondary outcomes, specifically evaluating how such factors were associated with responses to the primary outcome, with particular focus on clinical activity in cardiovascular or thoracic anaesthesiology (CVA). Additional secondary outcomes included self-reported understanding of specific CMR parameters and guidelines and respondents' perspectives on the limitations and future role of CMR in anaesthesia-related clinical decision making. All data were self-reported.

### Statistical analysis

The goal was to obtain as many responses as possible, with a predetermined minimal sample size of at least 300 responses. This target was based on feasibility estimates derived from anaesthesiologists' response rates reported in previously published surveys distributed by the same professional associations, accounting for potential reductions owing to factors such as topic and questionnaire fatigue.^17^, ^18^ Survey responses, even if incomplete, were included if the primary endpoint was answered, using an available-case analysis. As a result, the total number of responses (denominators) may vary across survey items (Supplementary Tables 1–7). Data are typically presented as frequencies (percentages rounded to the nearest integer). An ordinal logistic regression model was fitted with the response to the Likert-style questions as the ordinal outcome and the investigated factor (e.g. age, specialty) as the independent variable and reported with odds ratios (ORs) (95% confidence intervals [CIs]) (Supplementary Methods: statistics). The analyses were unadjusted. Multicollinearity statistics were assessed separately (Supplementary Table 8). A two-sided *P*-value of <0.05 was used to define statistical significance.

### Ethics and reporting standards

Participants provided consent by submitting the survey, and there were no incentives for completing it. As the survey was voluntary and anonymous, a regulatory waiver for its dissemination was granted by the Cantonal Ethics Committee Bern, Switzerland (Req. 2024-00548). This study was conducted and reported in accordance with the Strengthening the Reporting of Observational Studies in Epidemiology (STROBE)^32^ statement and the checklist for reporting results of internet e-surveys (CHERRIES).^33^

## Results

### Respondent characteristics

A total of 455 anaesthesiologists completed the survey for the primary endpoint, of 616 (74%) who opened the survey (Supplementary Fig 1). Most of the included respondents (84%) were board certified and from Europe (68%) (Supplementary Table 1). Just more than half (*n*=255, 56%) indicated CVA as an area of anaesthesia clinical activity (Supplementary Table 1). Before addressing the use of CMR as a perioperative risk assessment tool, respondents were asked which methods they routinely used for perioperative cardiac risk evaluation. Functional capacity was most frequently cited (*n*=425, 93%), followed by imaging in general terms (*n*=384, 84%), risk scores (*n*=354, 78%), stress tests (*n*=339, 75%), biomarkers (*n*=322, 71%), and angiography (*n*=274, 60%), which were less commonly used (Supplementary Table 2). When asked about local MRI infrastructure, 89% (*n*=403) reported having an MRI scanner in their local institute, and 65% (*n*=295) reported having on-site CMR services.

### Incorporation of cardiovascular magnetic resonance findings: primary endpoint

With regard to the primary endpoint, a total of 68% respondents (*n*=307) reported that they incorporated findings from CMR reports into their risk assessment when these reports were available in patient records: sometimes (*n*=48, 11%), usually (*n*=94, 21%), or almost always (*n*=165, 36%) (Fig 1). Conversely, 12% (*n*=53) rarely incorporated CMR findings, 6% (*n*=28) never did, and 15% (*n*=69) reported that CMR reports were not available at their institution (Supplementary Table 3). Among those who incorporated CMR findings, 55% (*n*=249) stated that they found the CMR results almost always or usually useful. Direct referral of patients to CMR by anaesthesiologists was rare, with only 5% (*n*=22) reporting having done so. A further 30% (*n*=138) had referred a patient for CMR after discussion with a cardiologist or radiologist (Supplementary Table 2).

### Factors associated with incorporation of cardiovascular magnetic resonance findings

**Fig 2:**
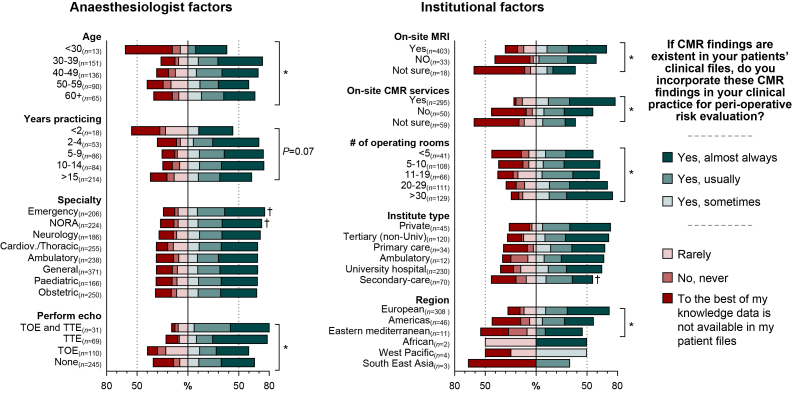
Factors associated with incorporation of clinical CMR findings. Red shades on the left represent low or no integration of CMR into perioperative risk evaluation, whereas green on the right represents its positive use. Dotted lines mark the 50% threshold, which, when crossed, indicates the view held by most respondents. Odds ratios for each analysis are presented in Supplementary Table 4. For single-choice questions: ∗*P*<0.05 significant difference among groups. For multi-choice questions (specialty and institute): ☨*P*<0.05 significant difference between answers if option selected or not. CMR, cardiovascular magnetic resonance imaging; NORA, non-operating room anaesthesia; TOE, transoesophageal echocardiography; TTE, transthoracic echocardiography.

Integration of CMR findings varied by both anaesthesiologist- and institution-related factors (Fig 2). For anaesthesiologist factors, younger respondents (<30 yr; *P*=0.03; OR per age category in Supplementary Table 4) were less likely to include CMR findings, with many in this group unaware of its availability in patient records. Across subspecialties, at least 50% reported incorporating findings from CMR reports. Performance of echocardiography was common (46%, *n*=210), with those who do transthoracic echocardiography more likely to incorporate CMR findings (OR 2.25 [1.37–3.71]; *P*=0.03).

Incorporation also varied with institutional characteristics, particularly local infrastructure. Respondents working in centres with MRI availability (*P*<0.01) (Fig 2, Supplementary Table 4) or where CMR was known to be performed (*P*<0.01) (Supplementary Table 4) were significantly more likely to incorporate CMR findings from reports in the patients' medical records. Notably, among anaesthesiologists from centres without access to MRI, 19 of 33 reported incorporating CMR findings. In addition, seven of 18 respondents who were unsure whether their centre had an MRI reported incorporating CMR findings. Working in hospitals with more operating rooms was associated with greater CMR use (*P*=0.02). However, the type of institution (i.e. university hospital, private hospital) was not significantly associated with inclusion of CMR findings.

### Specialisation in cardiovascular or thoracic anaesthesiology

Respondents who indicated they work in CVA were also more likely to be board-certified anaesthesiologists and to work in tertiary (university and non-university) hospitals than non-CVA colleagues, but there was no difference in age or years of practice (Supplementary Table 1). Responses to the primary question regarding incorporation of CMR findings from diagnostic reports were similar regardless of CVA involvement (CVA 69% *vs* non-CVA 67%; OR 1.17 [95% CI 0.84–1.63]; *P*=0.36) (Supplementary Table 2). However, CVA specialists were more likely to report understanding of the standard reporting in clinical CMR reports (*P*=0.02) (Supplementary Table 5).

**Fig 3:**
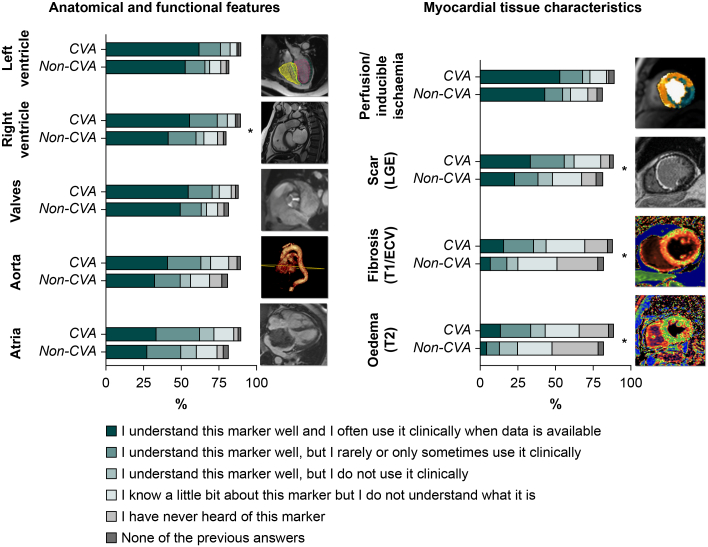
Which CMR features do anaesthesiologists understand and use? Most anaesthesiologists understand and use common ventricular findings but are unaware of or do not implement CMR measurements of myocardial tissue injury, which are key strengths of CMR, as indicated by lower percentages of green fields. In particular, assessments for diffuse fibrosis and oedema, representing chronic and acute injury respectively, are rarely understood. Odds ratios for each analysis are presented in Supplementary Table 7. ∗*P*<0.05 significant difference between anaesthesiologists with (top row) and without (bottom row) clinical activity in CVA. CMR, cardiovascular magnetic resonance imaging; CVA, cardiovascular or thoracic anaesthesiology; ECV, extracellular volume; LGE, late gadolinium enhancement (also known as delayed enhancement).

Further questions then specifically asked anaesthesiologists about individual parameters typically found in clinical CMR reports (Supplementary Table 7). Regarding specific CMR parameters, left ventricular anatomy and function were the parameters most frequently understood and used often (CVA 52%; non-CVA 53%) or at least sometimes (CVA 14%; non-CVA 13%) when available, with no difference based on CVA specialisation (OR 1.30 [95% CI 0.85–1.99]; *P*=0.23) (Fig 3). Other functional and anatomical parameters (ventricles, valves, aorta, atria) were used at least occasionally by >50% of participants in both groups, with responses similar between groups. The exception was right ventricular function, for which CVA respondents reported higher understanding and utilisation (often used; CVA 56%; non-CVA 42%; OR 1.65 [95% CI 1.11–2.47]; *P*=0.02). For tissue characterisation, inducible ischaemia by myocardial perfusion was the most frequently cited parameter (often used; CVA 53%; non-CVA 43%; OR 1.47 [95% CI 0.98–2.19]; *P*=0.06). Understanding and use of late gadolinium enhancement were significantly higher in CVA respondents (often used; CVA 34%; non-CVA 23%; OR 1.66 [95% CI 1.14–2.41]; *P*<0.01). Knowledge of advanced quantitative CMR techniques remained limited overall, but significantly higher in CVA: diffuse fibrosis (often used; CVA 16% *vs* non-CVA 7%; OR 2.33 [95% CI 1.60–3.40]; *P*<0.01) and myocardial oedema (often used; CVA 14% *vs* non-CVA 5%; OR 2.42 [95% CI 1.65–3.53]; *P*<0.01).

### Familiarity with guidelines

**Fig 4:**
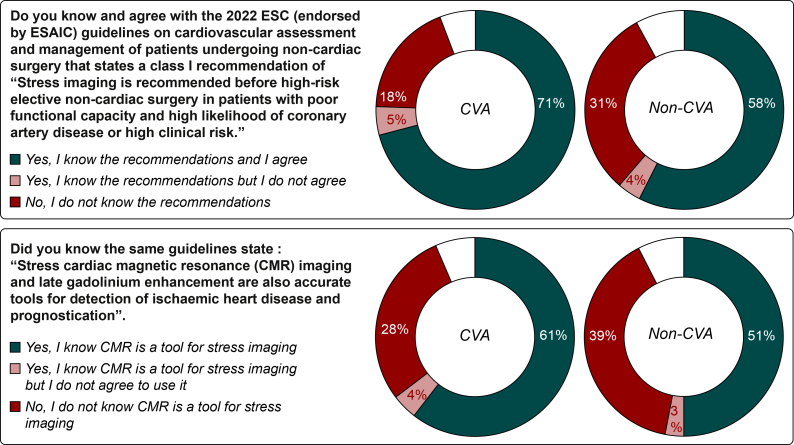
Familiarity with CMR in preoperative guidelines. Although anaesthesiologists were aware that stress imaging is recommended by the 2022 ESC guidelines endorsed by the ESAIC, anaesthesiologists with clinical activity in CVA were more likely to know this aspect (*P*<0.01, top). Moreover, there was a non-significant trend (*P*=0.06) for CVA anaesthesiologists to be more aware of the statements specifically concerning CMR (bottom). CMR, cardiovascular magnetic resonance imaging; CVA, cardiovascular or thoracic anaesthesiology; ESAIC, European Society of Anaesthesiology and Intensive Care; ESC, European Society of Cardiology.

Awareness of the 2022 ESC guidelines stating that ‘Stress imaging is recommended before high-risk elective noncardiac surgery in patients with poor functional capacity and high likelihood of coronary artery disease or high clinical risk’ was generally high. Notably, 70% were familiar with the recommendation and expressed agreement. Awareness was higher among CVA anaesthesiologists (CVA 71% *vs* non-CVA 58%; *P*=0.07) (Fig 4). When asked about the statement that ‘Stress cardiac magnetic resonance (CMR) imaging and late gadolinium enhancement are also accurate tools for detection of ischaemic heart disease and prognostication’, 61% of CVA *vs* 51% of non-CVA respondents indicated awareness (*P*=0.06).

### Future perspectives: cardiovascular magnetic resonance

**Fig 5:**
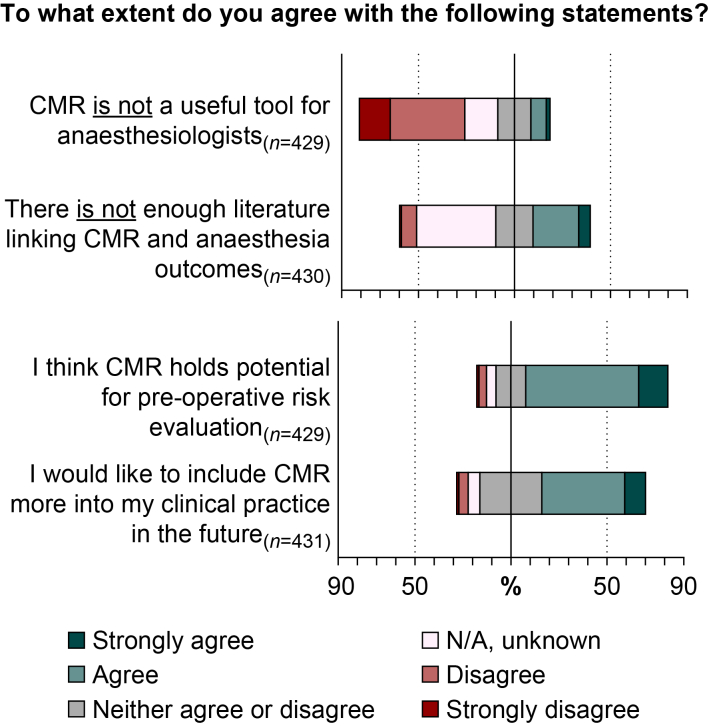
Current limitations and future perspectives of CMR. Most respondents recognised the potential of CMR for preoperative risk evaluation and expressed interest in future integration into their clinical practice, while also indicating limited existing literature linking CMR to anaesthesia outcomes. CMR, cardiovascular magnetic resonance imaging; N/A, not available.

Attitudes toward CMR were broadly positive (Fig 5). Only 10% agreed with the statement that ‘CMR is not a useful tool for anaesthesiologists’. Most (74%) believed that CMR has potential in perioperative risk assessment, and 54% expressed interest in greater integration into their clinical practice in the future. However, most acknowledged the lack of evidence linking CMR parameters to anaesthesia-related outcomes, with only 9% disagreeing (Supplementary Table 6).

## Discussion

In this online survey conducted in 2024–5, 68% of anaesthesiologists from 32 different countries reported incorporating findings from CMR reports for their perioperative risk assessments, at least sometimes, when data were available in patients' medical records. Anaesthesiologists indicated that CMR holds potential for perioperative risk evaluation; however, there is currently insufficient evidence linking CMR findings to anaesthesia-related outcomes.

### Who is incorporating cardiovascular magnetic resonance findings into their risk evaluations?

The survey was not aimed to assess whether anaesthesiologists request or independently interpret CMR studies, but rather to determine who uses structured clinical report data in perioperative decision-making. It also did not explore how anaesthesiologists were to use CMR data (e.g. to postpone surgery or request additional tests); rather, it aimed to provide initial insights into whether CMR data were used for any purpose in perioperative risk evaluation. Identifying which anaesthesiologists engage with CMR findings can help guide targeted educational strategies and foster collaborations with imaging specialists, thereby supporting broader use of CMR. Anaesthesiologists from centres with more operating rooms and on-site CMR facilities reported a higher incorporation of CMR findings, likely reflecting a greater proportion of high-risk patients and diagnostic facilities at these institutions. Nevertheless, among respondents without in-house MRI, many still said that they incorporated CMR findings when available. Diagnostic imaging can be performed externally, with reports subsequently sent to the operating hospital along with the patient's remainingrecords. Therefore, CMR findings may be available despite the absence of on-site services. In contrast to initial assumptions, institute type did not seem to be univariately associated with responses; anaesthesiologists working in university or tertiary hospitals did not report higher inclusion of CMR data than those in other settings. Although university hospitals are typically high-volume CMR centres, a 2023 survey showed that 25% of community or non-academic hospitals also had CMR programmes, indicating that direct access is no longer confined to tertiary centres.^19^ Interestingly, younger anaesthesiologists were the least likely to incorporate CMR findings. Of the respondents younger than 30 yr or with <2 years of clinical experience, 89% worked in university or tertiary hospitals, which may suggest that the barrier is not access but limited endorsement of guidelines and training. It has been reported that younger physicians tend to adhere strictly to guidelines,^20^^,^^21^ and CMR is rarely mentioned in current recommendations. Notably, anaesthesiologists without CVA subspecialisation also reported using CMR findings in noncardiac surgical patients, emphasising that interdisciplinary collaboration should extend beyond cardiovascular surgery alone. As this survey did not examine how CMR influenced perioperative risk assessment, future studies are warranted to provide more in-depth analyses into the impact of CMR use.

### Multiparametric nature of cardiovascular magnetic resonance reports

Although the overall incorporation of CMR findings into perioperative risk assessment was higher than anticipated, anaesthesiologists predominantly relied on conventional parameters, such as ventricular function and valve assessment, which are readily available on echocardiography. In contrast, understanding of advanced tissue characterisation markers, for which CMR offers a distinct diagnostic advantage (including myocardial fibrosis and oedema), was limited, particularly among non-CVA anaesthesiologists. Right ventricular function, despite its prognostic relevance in both cardiac and noncardiac surgery,^22^^,^^23^ was also less frequently recognised. Guidelines from American societies acknowledge CMR as the reference standard for quantitative right ventricular assessment in selected patients,^24^ yet its application remains limited.

Traditional tissue-based measurements, such as stress perfusion to assess inducible ischaemia and late gadolinium enhancement for detecting myocardial scar, have been mainstays in clinical reports for more than 2 decades. In fact, these are the two sequences addressed in the 2022 ESC guidelines on cardiovascular assessment and management of patients undergoing noncardiac surgery for the detection of ischaemic heart disease.^15^ However, they are not included among the higher-class recommendations in current guidelines for perioperative cardiovascular risk assessment, nor is there robust literature on the topic. Consequently, even when anaesthesiologists consult cardiology or radiology imaging specialists, any decisions on CMR-based perioperative management plans are not evidence-based. More than half of surveyed anaesthesiologists reported understanding and using myocardial perfusion/inducible ischaemia measurements, with no difference between non-CVA and CVA specialists. Abnormal myocardial perfusion assessed using nuclear or CMR stress imaging has been associated with poor postoperative outcomes at 30 and 180 days after surgery,^8^^,^^25^ yet the evidence for stress imaging in preoperative risk stratification is limited and has not been adequately investigated^24^; thus, the 2022 guidelines^15^ still state ‘the impact of stress imaging (echocardiography or CMR) before noncardiac surgery on reduction of perioperative cardiovascular complications in non-ischaemic heart diseases needs further research’.

Late gadolinium enhancement imaging, which reveals chronic injury and tissue viability, represents another long-established CMR marker. It has also been shown to be one of the strongest predictors of long-term survival after coronary artery bypass graft surgery and has been suggested to improve preoperative risk assessment for long-term prognosis.^26^ However, the understanding of scar imaging declines, especially among non-CVA anaesthesiologists.

### Modern markers in cardiovascular magnetic resonance reporting

A major CMR advancement over the past 15 years is parametric mapping for quantifying myocardial injury, including T1 and T2 imaging for oedema and T1/extracellular volume imaging for diffuse fibrosis. At the time of writing this paper, these parameters are not included in existing perioperative guidelines but are incorporated into CMR imaging and reporting guidelines.^27^^,^^28^ However, emerging evidence suggests potential relevance to perioperative and intensive care medicine.^29^ In a case series, T2 imaging detected acute myocardial injury in patients with troponin-defined myocardial injury after noncardiac surgery.^30^ Moreover, parametric maps can distinguish chronic from acute injury, offering insight into the current stage of myocardial injury, which may be relevant as systemic inflammation is associated with perioperative myocardial injury.^31^Knowledge of these markers within the community remains limited; moreover, their adoption may be influenced by the sheer number of available CMR parameters, making it difficult for non-imaging specialists to appreciate the specific benefits of each marker.

### Research and clinical implications

The findings highlight the expanding clinical reach of CMR beyond traditional medical fields. The interest in CMR expressed by anaesthesiologists who responded to this survey contrasts with the limited literature addressing its perioperative application. Although most anaesthesiologists agreed that there was insufficient literature linking CMR to perioperative outcomes, they perceived potential for its future use in the field. Therefore, further studies are needed to determine whether CMR-derived parameters can improve perioperative risk stratification beyond standard tools such as echocardiography, biomarkers, and clinical risk scores. If studies find evidence that CMR has prognostic potential for anaesthesiologists, targeted educational initiatives could equip anaesthesiologists with the knowledge and skills to interpret CMR findings and incorporate them into risk assessments. Together, these efforts could address current knowledge gaps and support evidence-based adoption of CMR into anaesthetic practice. Leveraging CMR data already available in medical records may further enable more cost-efficient preoperative evaluation compared with the routine acquisition of additional standard tests.

### Limitations and generalisability

This was an opt-in web-based survey that relied on non-probability sampling rather than random sampling. Therefore, the high proportion of positive views on CMR should be interpreted with caution owing to potential self-selection bias, as physicians with greater interest in CMR or cardiac imaging may have been more likely to participate. Moreover, in addition to broad-based anaesthesia societies, the European Association of Cardiothoracic Anaesthesia also distributed the survey, likely leading to a higher response rate from CVAs. These factors could have led to overestimation of overall familiarity and use of CMR and distorted associations observed in subgroup analyses. Geographic representation was unequal, with most respondents from Europe, especially Switzerland, likely because more European and Swiss societies agreed to distribute the survey. Response rates for some outcomes were sparse, and the smaller sample size did not allow for assessment of interaction effects. Analyses were univariable, and the reported ORs were unadjusted for inter-related factors. Thus, these survey findings should be regarded as exploratory rather than as concrete independent determinants of CMR inclusion. These findings may not be representative of all regions. As the survey remained open for an extended recruitment period and no technical duplicate-detection measures were used, duplicate participation could not be formally excluded. Only an English version was provided, and potential misunderstandings by respondents can introduce error. Finally, only anaesthesiologists were invited to participate, so trends among non-physician anaesthesia personnel were not captured. Response rate and view rate cannot be calculated as it is unknown how many anaesthesiologists received the link.

### Conclusion

Although the link between CMR and perioperative outcomes has not yet been systematically established, this voluntary survey shows that, among the responding anaesthesiologists, CMR data are already integrated into their perioperative risk assessment when available. Even anaesthesiologists not specialising in cardiovascular anaesthesia incorporate CMR results. Further outcome-driven research combined with targeted education and interdisciplinary collaboration is needed to support evidence-based integration of CMR into anaesthetic practice. Importantly, this survey suggests that anaesthesiologists are receptive to adopting CMR into their clinical workflow once sufficient evidence becomes available.

## Authors’ contributions

Conceptualisation: KF, RH, DPG

Methodology: KF, RH, TS, DPG

Data curation: KF, RH, JNF

Formal analysis: KF, RH, JNF

Visualisation: KF, RH

Writing—original draft: KF, RH, DPG

Writing—review and editing: all authors

Supervision: KF, DPG

## Data availability statement

De-identified survey data are available from the corresponding author upon reasonable request, subject to institutional data protection policies

## Declaration of generative AI and AI-assisted technologies in the writing process

During the preparation of this work, the authors used OpenAI ChatGPT (GPT-5.1) to assist with grammar and language editing of manuscript text originally written by the authors. After using this tool/service, the authors reviewed and edited the content as needed and take full responsibility for the published article.

## Funding

Bern University Hospital Department of Anaesthesiology and Pain Medicine scientific funds (FIFK-25-1).

## Declarations of interest

The authors declare that they have no conflicts of interest.

## References

[1] Kristensen S.D., Knuuti J., Saraste A. (2014). 2014 ESC/ESA Guidelines on non-cardiac surgery: cardiovascular assessment and management: the Joint Task Force on non-cardiac surgery: cardiovascular assessment and management of the European Society of Cardiology (ESC) and the European Society of Anaesthesiology (ESA). Eur Heart J.

[2] Smilowitz N.R., Redel-Traub G., Hausvater A. (2019). Myocardial injury after noncardiac surgery: a systematic review and meta-analysis. Cardiol Rev.

[3] Knoedler S., Matar D.Y., Friedrich S. (2023). The surgical patient of yesterday, today, and tomorrow—a time-trend analysis based on a cohort of 8.7 million surgical patients. Int J Surg.

[4] Smilowitz N.R., Gupta N., Guo Y., Beckman J.A., Bangalore S., Berger J.S. (2018). Trends in cardiovascular risk factor and disease prevalence in patients undergoing non-cardiac surgery. Heart.

[5] Timmis A., Aboyans V., Vardas P. (2024). European Society of Cardiology: the 2023 atlas of cardiovascular disease statistics. Eur Heart J.

[6] Rodseth R.N., Lurati Buse G.A., Bolliger D. (2011). The predictive ability of pre-operative B-type natriuretic peptide in vascular patients for major adverse cardiac events: an individual patient data meta-analysis. J Am Coll Cardiol.

[7] Cohn S.L., Fernandez Ros N.F. (2018). Comparison of 4 cardiac risk calculators in predicting postoperative cardiac complications after noncardiac operations. Am J Cardiol.

[8] Sukprasert N., Wanitchung K., Prechawuttidech S., Tongsai S., Kaolawanich Y. (2025). Clinical utility of preoperative stress perfusion cardiac magnetic resonance for predicting cardiovascular events in patients undergoing major noncardiac surgery. Ann Med.

[9] Weiner J., Heinisch C., Oeri S. (2023). Focal and diffuse myocardial fibrosis both contribute to regional hypoperfusion assessed by post-processing quantitative-perfusion MRI techniques. Front Cardiovasc Med.

[10] O’Brien A.T., Gil K.E., Varghese J., Simonetti O.P., Zareba K.M. (2022). T2 mapping in myocardial disease: a comprehensive review. J Cardiovasc Magn Reson.

[11] Ordovas K.G., Baldassarre L.A., Bucciarelli-Ducci C. (2021). Cardiovascular magnetic resonance in women with cardiovascular disease: position statement from the Society for Cardiovascular Magnetic Resonance (SCMR). J Cardiovasc Magn Reson.

[12] Fischer K., Becker P., Mongeon F.-P. (2022). Feature tracking strain analysis detects the onset of regional diastolic dysfunction in territories with acute myocardial injury induced by transthoracic electrical interventions. Sci Rep.

[13] Grob L., Schwerzmann Y., Kaiser D. (2025). Retrospective temporal resolution interpolation alters myocardial strain quantification on compressed sensing cine CMR. Int J Cardiovasc Imaging.

[14] Cohen Y.A., Bremner L., Shetty M. (2025). Temporal trends in noninvasive and invasive cardiac testing from 2010 to 2022 in the US medicare population. Circ Cardiovasc Imaging.

[15] Halvorsen S., Mehilli J., Cassese S. (2022). 2022 ESC Guidelines on cardiovascular assessment and management of patients undergoing non-cardiac surgery: developed by the task force for cardiovascular assessment and management of patients undergoing non-cardiac surgery of the European Society of Cardiology (ESC): endorsed by the European Society of Anaesthesiology and Intensive Care (ESAIC). Eur Heart J.

[16] Harris P.A., Taylor R., Thielke R., Payne J., Gonzalez N., Conde J.G. (2009). Research Electronic Data Capture (REDCap)—a metadata-driven methodology and workflow process for providing translational research informatics support. J Biomed Inform.

[17] Mackert S., Walker M., Pirlich N. (2025). Current airway management practice among Swiss anesthesiologists: results of a national survey. Anaesthesiologie.

[18] Paternoster G., Sangalli F., de Arroyabe B.M.L. (2024). Insights into perioperative hypertension management in Europe: results from a survey endorsed by the European association of cardiothoracic anaesthesiology and intensive care (EACTAIC). J Cardiothorac Vasc Anesth.

[19] Sierra-Galan L.M., Estrada-Lopez E.E.S., Ferrari V.A. (2023). Worldwide variation in cardiovascular magnetic resonance practice models. J Cardiovasc Magn Reson.

[20] Hoorn C.J.G.M., Crijns H.J.G.M., Dierick-van Daele A.T.M., Dekker L.R.C. (2019). Review on factors influencing physician guideline adherence in cardiology. Cardiol Rev.

[21] Hassan S., Chan V., Stevens J., Stupans I. (2021). Factors that influence adherence to surgical antimicrobial prophylaxis (SAP) guidelines: a systematic review. Syst Rev.

[22] Alavi N., Van Klei W., Agyei K. (2025). The association of right ventricular function with outcomes after cardiac surgery: a systematic review. Can J Anaesth.

[23] Chou J., Ma M., Gylys M. (2019). Preexisting right ventricular dysfunction is associated with higher postoperative cardiac complications and longer hospital stay in high-risk patients undergoing nonemergent major vascular surgery. J Cardiothorac Vasc Anesth.

[24] Thompson A., Fleischmann K.E., Smilowitz N.R. (2024). 2024 AHA/ACC/ACS/ASNC/HRS/SCA/SCCT/SCMR/SVM guideline for perioperative cardiovascular management for noncardiac surgery: a report of the American College of Cardiology/American Heart Association joint committee on clinical practice guidelines. Circulation.

[25] Fazzari F., Lisi C., Catapano F. (2024). Prognostic value of stress CMR and SPECT-MPI in patients undergoing intermediate-to-high-risk non-cardiac surgery. Radiol Med.

[26] T Yang, M Lu, W Ouyang (2021). Prognostic value of myocardial scar by magnetic resonance imaging in patients undergoing coronary artery bypass graft. Int J Cardiol.

[27] Hundley W.G., Bluemke D.A., Bogaert J. (2022). Society for Cardiovascular Magnetic Resonance (SCMR) guidelines for reporting cardiovascular magnetic resonance examinations. J Cardiovasc Magn Reson.

[28] Kramer C.M., Barkhausen J., Bucciarelli-Ducci C., Flamm S.D., Kim R.J., Nagel E. (2020). Standardized cardiovascular magnetic resonance imaging (CMR) protocols: 2020 update. J Cardiovasc Magn Reson.

[29] Isaak A., Pomareda I., Mesropyan N. (2023). Cardiovascular magnetic resonance in survivors of critical illness: cardiac abnormalities are associated with acute kidney injury. J Am Heart Assoc.

[30] Guensch D.P., Friess J.O., Stiffler S. (2024). Visualising myocardial injury after noncardiac surgery: a case series using postoperative cardiovascular MRI. Br J Anaesth.

[31] Ackland G.L., Abbott T.E.F., Cain D. (2019). Preoperative systemic inflammation and perioperative myocardial injury: prospective observational multicentre cohort study of patients undergoing non-cardiac surgery. Br J Anaesth.

[32] von Elm E, Altman DG., Egger M., Pocock SJ., Gøtzsche PC., Vandenbroucke JP. (2007). STROBE Initiative. The Strengthening the Reporting of Observational Studies in Epidemiology (STROBE) statement: guidelines for reporting observational studies. Bull World Health Organ.

[33] Eysenbach (2004). G, Improving the quality of web surveys: the checklist for reporting results of internet E-surveys (CHERRIES). J Med Internet Res.

