# Supplementary material for: A cross-sectional online survey evaluating how anaesthesiologists view cardiovascular magnetic resonance imaging reports as a tool for perioperative risk evaluation
Source: BJA Open. 2026 Jul 31;19:100649. doi: 10.1016/j.bjao.2026.100649 (PMC13452394; doi:10.1016/j.bjao.2026.100649)
Supplement: Supplementary file 1 [file mmc1.pdf]

SUPPLEMENTAL INFORMATION

A cross-sectional online survey evaluating how anaesthesiologists view cardiovascular magnetic resonance imaging reports as a tool for perioperative risk evaluation

Table of Contents

SUPPLEMENTAL METHODS: SURVEY INVITATIONS 2

SUPPLEMENTAL METHODS: STATISTICS 3

SUPPLEMENTAL FIGURE 1: RESPONSE FLOW DIAGRAM 4

SUPPLEMENTAL TABLE 1: RESPONDER CHARACTERISTICS 5

SUPPLEMENTAL TABLE 2: AVAILABILITY OF CMR AND AWARENESS OF THE GUIDELINES 7

SUPPLEMENTAL TABLE 3: COUNTRIES WHO DO NOT HAVE ACCESS TO CMR, OR DO NOT HAVE CMR FINDINGS AVAILABLE IN THE CLINICAL FILES OF THEIR PATIENTS 10

SUPPLEMENTAL TABLE 4: ODDS RATIO'S FOR FIGURE 2 INVESTIGATING HOW PERSONAL AND INSTITUTIONAL FACTORS INFLUENCE THE RESPONSE TO THE PRIMARY ENDPOINT 11

SUPPLEMENTAL TABLE 5: ACCESS AND UNDERSTANDING OF CLINICAL CMR REPORTS 13

SUPPLEMENTAL TABLE 6: OPINIONS ON CMR LITERATURE AND USEFULNESS 14

SUPPLEMENTAL TABLE 7: USE OF INDIVIDUAL CMR PARAMETERS 15

SUPPLEMENTAL TABLE 8. ASSESSMENT OF MULTICOLLINEARITY 20

Supplemental Methods: Survey Invitations

Sample text for survey distribution

Dear colleagues

As anaesthesiologists, we are seeing a rising number of patients with multiple cardiac risk factors in our daily practice, and these patients come with a high risk for peri-operative adverse events. **Cardiovascular magnetic resonance (CMR)** imaging**,** also known as cardiac MRI, is a non-invasive imaging technique that can quantify multiple parameters of the heart chambers and tissue along with surrounding vasculature.

We are reaching out to anaesthesiologists internationally to participate in an online survey to understand if and how you incorporate findings from modern diagnostic imaging techniques, especially **CMR** imaging reports, into your **peri-operative risk evaluation**.

This survey has been endorsed by the European Association of Cardiothoracic Anaesthesiology and Intensive Care (EACTAIC) and by the Swiss Society for Anaesthesiology and Perioperative Medicine (SSAPM). It is very brief and should take less than 10minutes of your time. You can find the link to the online survey here:

https://redcap.link/survey_anaesthesiology_cmr

*(*link deactivated following survey closure)*

Thank you very much in advance for your time and cooperation, your feedback is very important to us. Further, please feel free to distribute the survey among colleagues.

Best regards,

Dr. Kady Fischer,
Dr. Raul Harms,
Prof. Dr. Dominik Guensch

INSELSPITAL
Department of Anaesthesiology and Pain Medicine
Inselspital, Bern University Hospital, University of Bern,
3010 Bern
Switzerland
www.anaesthesie.insel.ch

Supplemental Methods: Statistics

For the statistical models, survey responses, even if incomplete, were included if the primary endpoint was answered, using an available-case analysis. Additionally, responses were not included in the statistical analysis for questions which had the options “None of the previous answers” or “N/A, unknown”. As a result, total responses may vary across survey item. However, for all of the above, the frequency of responses these options obtained was reported in the tables and figures

Supplemental Figure 1: Response Flow Diagram

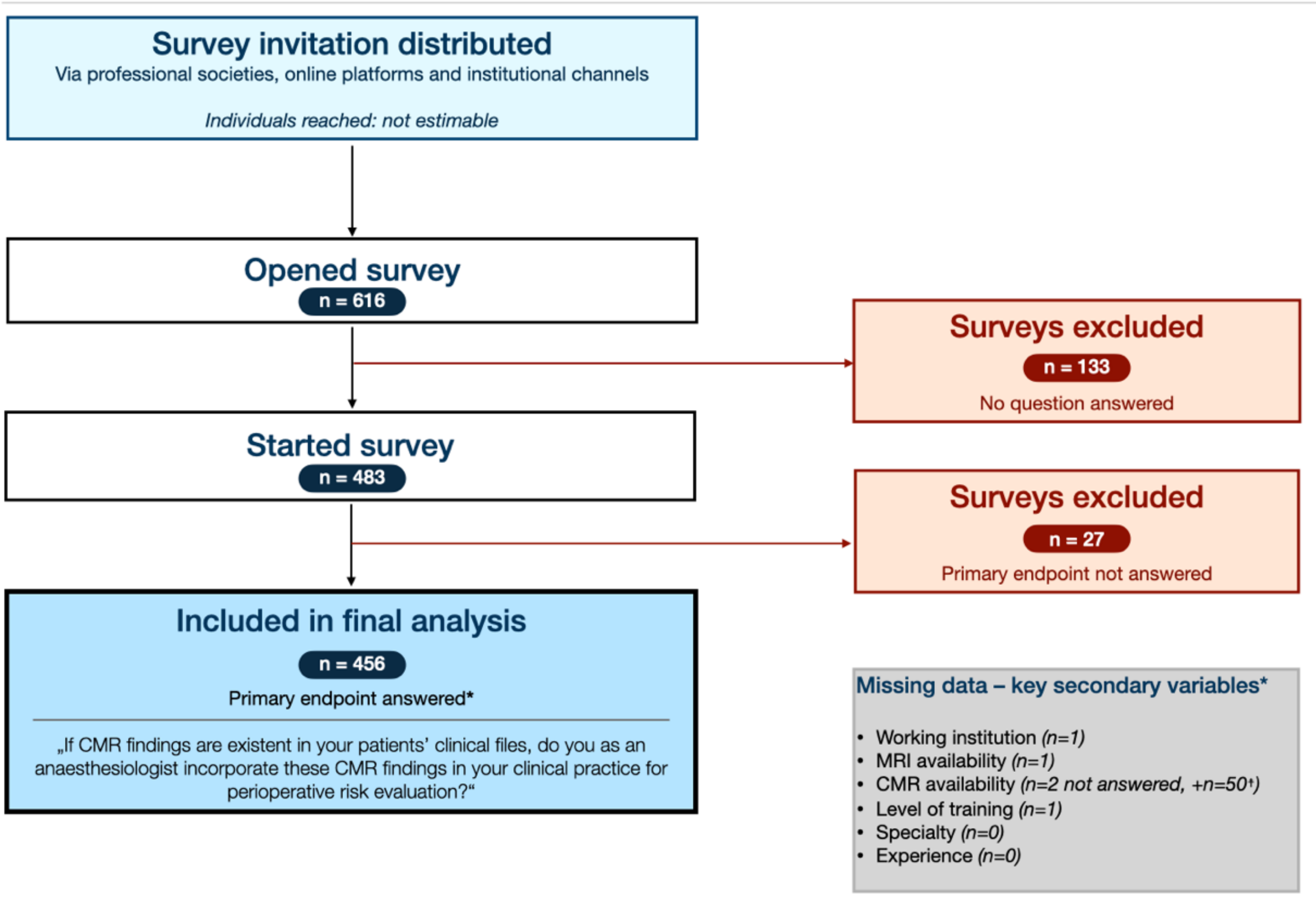

* Incomplete responses retained for analysis; missing data for key secondary variables are listed above.

†Local CMR availability was only shown to participants who answered they had MRI availability

Supplemental Table 1: Responder Characteristics

		Frequency (%)			Statistical Model		
**Question**	**Responses**	**All (n=455)**	**CVA (n=255)**	**Non-CVA (n=200)**	**OR (95%-CI)**	**#Obs**	**p-value**
Age, years	<30	13 (3%)	7 (3%)	6 (3%)	0.86 *(0.53-1.39)*	455	0.54
	30-39	151 (33%)	87 (34%)	64 (32%)			
	40-49	136 (30%)	70 (28%)	66 (33%)			
	50-59	90 (20%)	56 (22%)	34 (17%)			
	>60	65 (14%)	35 (54%)	30 (15%)			
Level of training	Resident	73 (16%)	37 (15%)	36 (18%)	0.75 *(0.61-0.92)*	454	<0.01
	Board certified	381 (84%)	218 (86%)	163 (82%)			
	Not answered	1 (<1%)	0	1 (<1%)			
Experience, years	<2	18 (4%)	3 (1%)	15 (8%)	0.82 *(0.58-1.16)*	455	0.26
	2-4	53 (12%)	33 (13%)	20 (10%)			
	5-9	86 (19%)	48 (19%)	38 (19%)			
	10-14	84 (19%)	47 (18%)	37 (19%)			
	>15	214 (47%)	124 (49%)	90 (45 %)			
Areas of anaesthesia clinical activity*	Ambulatory anaesthesia	238 (52%)					
	Cardio-, vascular-, thoracic anaesthesiology	255 (56%)					
	Emergency medicine	206 (45%)					
	General anaesthesiology	371(82%)					
	Neuro-anaesthesia	186 (41%)					
	No operation-room anaesthesia	224 (49%)					
	Obstetric anaesthesiology	250 (55%)					
	Pediatric-/neonatal anaesthesiology	166 (37%)					
	Other	56 (12%)					
Type of institution*	University hospital	230 (51%)	144 (57%)	86 (43%)	1.72 *(1.18-2.52)*	455	<0.01
	Non-university tertiary care hospital	120 (26%)	83 (33%)	37 (19%)	2.13 *(1.36-3.31)*	455	<0.01
	Secondary care hospital	70 (15%)	26 (10%)	44 (22%)	0.40 *(0.24-0.68)*	455	<0.01
	Primary care hospital	34 (8%)	10 (4%)	24 (12%)	0.30 *(0.14-0.64)*	455	<0.01
	Private hospital	45 (10%)	28 (11%)	17 (9%)	1.32 *(0.70-2.50)*	455	0.38
	Ambulatory anaesthesia provider	12 (3%)	5 (2%)	7 (4%)	0.55 *(0.17-1.76)*	455	0.31
	Other	4 (<1%)	1 (<1%)	3 (2%)	-	-	-
Number of operating rooms, n	<5	41 (9%)	8 (3%)	33 (17%)	2.26 *(1.61-3.16)*	455	<0.01
	5-10	108 (24%)	49 (19%)	59 (30%)			
	11-19	66 (14%)	43 (17%)	23 (12%)			
	20-29	111 (24%)	74 (29%)	37 (19%)			
	>30	129 (28%)	81 (32%)	48 (24%)			
Number of anaesthesia	< 1000	13 (3%)	4 (2%)	9 (5%)	2.00	454	<0.01
	1000-3499	47 (10%)	17 (7%)	30 (15%)			

procedures performed yearly, n	3500-7499	67 (15%)	28 (11%)	39 (20%)	*(1.44-2.79)*		
	7500-12499	62 (14%)	38 (15%)	24 (12%)			
	12500-19999	49 (11%)	31 (12%)	18 (9%)			
	>20000	129 (28%)	79 (31%)	50 (25%)			
	Unknown	87 (19%)	57 (22%)	30 (15%)			
	Not answered	1 (<1%)	1 (<1%)	0			
World Health Organization Defined Region	African region<br>- South Africa<br>- Uganda	2 (<1%)	1 (<1%)	1 (<1%)			
	Eastern Mediterranean region<br>- Egypt<br>- Kuwait<br>- Lebanon<br>- Oman<br>- Palestinian Territories<br>- Saudi Arabia	11 (2%)	9 (4%)	2 (1%)			
	European region<br>- Switzerland<br>- Belgium<br>- Croatia<br>- Denmark<br>- France<br>- Germany<br>- Greece<br>- Ireland<br>- Italy<br>- Latvia<br>- Netherlands<br>- Portugal<br>- Spain<br>- Sweden<br>- Turkey<br>- United Kingdom	308 (68%)	167 (81%)	141 (84%)			
	Regions of the Americas<br>- Canada<br>- Mexico<br>- United States of America	46 (10%)	24 (12%)	22 (13%)			
	Southeast Asia Region<br>- India<br>- Nepal	3 (<1%)	2 (1%)	1 (<1%)			
	Western Pacific Region<br>- Australia<br>- Malaysia	4 (<1%)	3 (2%)	1 (<1%)			
	Not answered/not listed	81 (18%)	49 (19%)	32 (16%)			

Frequencies (percentages) are reported * Multiple responses allowed. For each region, the countries of the anaesthesiologists who responded are indicated.

Supplemental Table 2: Availability of CMR and Awareness of the Guidelines

		Frequency (%)			Statistical Model		
**Question**	**Responses**	**All (n=455)**	**CVA (n=255)**	**Non-CVA (n=200)**	**OR (95% -CI)**	**#Obs**	**p-value**
**Which pre-operative cardiac risk-assessment tools do you as an anaesthesiologist use?** (multiple answers possible)	Risk scores *(Revised Cardiac Risk Index, Surgical Risk Calculator, American College of Surgeons - National Surgical Quality Improvement Program)*	354 (78%)	200 (78%)	154 (77%)	1.09 *(0.70-1.70)*	455	0.72
	Functional capacity *(based on in-/ability to climb two flights of stairs, Duke Activity Status Index or cardiopulmonary exercise testing)*	425 (93%)	238 (93%)	187 (94%)	0.97 *(0.46-2.05)*	455	0.94
	Biomarkers *(cardiac troponins, BNP/NT-BNP)*	322 (71%)	189 (74%)	133 (67%)	1.44 *(0.96-2.17)*	455	0.08
	Angiography *(coronary computed tomography, invasive angiography)*	274 (60%)	179 (70%)	95 (48%)	2.60 *(1.77-3.83)*	455	<0.001
	Imaging *(transthoracic echocardiogram, transoesophageal echocardiogram, cardiovascular magnetic resonance imaging, nuclear imaging)*	384 (84%)	221 (87%)	163 (82%)	1.48 *(0.89-2.45)*	455	0.84
	Stress Tests *(exercise stress test, dobutamine stress, echocardiography, stress cardiac magnetic resonance)*	339 (75%)	194 (76%)	145 (73%)	1.21 *(0.79-1.84)*	455	0.39
**Does your hospital have a clinical magnetic resonance imaging scanner (MRI)?**	Yes	403 (89%)	238 (93%)	165 (83%)	3.42 *(1.81-6.46)*	454	<0.01
	No	33 (7%)	12 (5%)	21 (11%)			
	I am not sure	18 (4%)	4 (2%)	14 (7%)			
	Not Answered	1 (<1%)	1 (<1%)	0 (0%)			
**Does your hospital perform CMR?**	Yes	295 (65%)	192 (75%)	103 (52%)	2.34 *(1.51-3.65)*	404	<0.01
	No	49 (11%)	19 (8%)	30 (15%)			
	I am not sure	59 (13%)	27 (11%)	32 (16%)			
	Not Answered†	51 (11%)	16 (6%)	35 (18%)			
**<u>If</u> CMR findings are existent in your patients' clinical files, do you as an anaesthesiologist incorporate these CMR findings in your clinical practice for peri-operative risk evaluation?**	Yes, almost always	165 (36%)	92 (36%)	73 (37%)	1.17 *(0.84-1.63)*	455	0.36
	Yes, usually	94 (21%)	55 (22%)	39 (20%)			
	Yes, sometimes	48 (11%)	29 (11%)	19 (10%)			
	Rarely	53 (12%)	37 (15%)	16 (8%)			
	No, never	26 (6%)	14 (6%)	12 (6%)			
	To the best of my knowledge, CMR findings are not available in the clinical files of my patients	69 (15%)	28 (11%)	41 (21%)			
**Primary Endpoint*	Not Answered	-	-	-			

		Frequency (%)			Statistical Model		
**Question**	**Responses**	**All (n=455)**	**CVA (n=255)**	**Non-CVA (n=200)**	**OR (95% -CI)**	**#Obs**	**p-value**
***If you responded yes to the previous question, did you find the CMR results useful?** (only available to participants who responded to the first four responses in previous question)	Yes, almost always	103/347 (30%)	62/213 (35%)	41/147 (31%)	1.15 *(0.78-1.70)*	347	0.48
	Yes, usually	146/347 (42%)	85/213 (48%)	61/147 (47%)			
	Yes, sometimes	79/347 (23%)	43/213 (24%)	36/147 (27%)			
	Rarely	18/347 (5%)	11/213 (6%)	7/147 (5%)			
	No, never	1/347 (<1%)	0 /213 (0%)	1/147 (<1%)			
	Not Answered	13/347 (4%)	12/213 (6%)	1/147 (<1%)			
**As an anaesthesiologist have you ever referred a patient to CMR for peri-operative risk evaluation?** (multiple answers possible)	Yes, I have directly referred a patient for a pre-operative CMR exam	22 (5%)	17 (7%)	5 (3%)	0.36 *(0.13-0.99)*	455	0.04
	Yes, I have discussed with a specialist (ie. cardiologist or radiologist) to refer a patient for a pre-operative CMR exam	138 (30%)	77 (30%)	61 (31%)	1.04 *(0.69-1.55)*	455	0.94
	No, none of the above	296 (65%)	161 (63%)	135 (68%)	1.19 *(0.81-1.76)*	455	0.33
**Do you know and agree with the 2022 European Society of Cardiology guidelines (endorsed by the European Society of Anaesthesiology and Intensive Care) on cardiovascular assessment and management of patients undergoing non-cardiac surgery that states a class I recommendation of: "Stress imaging is recommended before high-risk elective non-cardiac surgery in patients with poor functional capacity and high likelihood of coronary artery disease or high clinical risk"?**	Yes, I know the recommendations and I agree	296 (65%)	181 (71%)	115 (58%)	1.77 *(1.17-2.68)*	424	<0.01
	No, I do not know the recommendations	108 (24%)	47 (18%)	61 (31%)			
	Yes, I know the recommendations but I do not agree	20 (4%)	12 (5%)	8 (4%)			
	Not Answered	31 (7%)	15 (6%)	16 (8%)			
**Do you know the same guidelines state: "Stress**	Yes, I know CMR is a tool for stress imaging	257 (57%)	156 (61%)	101 (51%)	1.46 *(0.99-2.15)*	422	0.06

		Frequency (%)			Statistical Model		
**Question**	**Responses**	**All (n=455)**	**CVA (n=255)**	**Non-CVA (n=200)**	**OR (95% -CI)**	**#Obs**	**p-value**
**cardiac magnetic resonance (CMR) imaging and late gadolinium enhancement are also accurate tools for detection of ischaemic heart disease and prognostication"?**	No, I do not know CMR is a tool for stress imaging	149 (33%)	72 (28%)	77 (39%)			
	Yes, I know CMR is a tool for stress imaging, but I do not agree to use it	16 (4%)	10 (4%)	6 (3%)			
	Not Answered	33 (7%)	17 (7%)	16 (8%)			

Frequencies (percentages) are reported. CVA: anaesthesiologists who indicated cardio-, vascular-, or thoracic anaesthesiology as an area of clinical expertise. From the statistical model, the adjusted odds ratio (OR) along with 95% confidence intervals and number of observations remaining in the final number (#Obs) are reported. An odds ratio greater than 1 indicates increased use by CVA.

*

†Local CMR availability was only shown to participants who answered they had MRI availability

Supplemental Table 3: Countries Who Do Not Have Access to CMR, or Do Not Have CMR Findings Available in the Clinical Files of Their Patients

Question	Country	n
**Answered "No" or "Not Sure" to "Does your hospital have a clinical magnetic resonance imaging scanner (MRI)?"**	Country not answered	12
	Switzerland	12
	Canada	6
	Greece	6
	United States of America	3
	Croatia	2
	Turkey	2
	Mexico	2
	France	1
	India	1
	Egypt	1
	Germany	1
	Nepal	1
	Ireland	1
**Answered "To the best of my knowledge, CMR findings are not available in the clinical files of my patients"**	Switzerland	20
	Country not answered	13
	Canada	7
	Turkey	6
	Greece	6
	United States of America	5
	Croatia	2
	Egypt	2
	Portugal	1
	Lebanon	1
	Latvia	1
	Germany	1
	India	1
	Malaysia	1
	Nepal	1
	Mexico	1

Supplemental Table 4: Odds Ratio's for Figure 2 Investigating How Personal and Institutional Factors Influence the Response to the Primary Endpoint

		Statistical Model		
**Ordinal Regression**	**Responses**	**OR (95%-CI)**	**#Obs**	**p-value**
**Age, years**	<30*	-	455	0.03
	30-39	4.27 *(1.54-11.8)*		
	40-49	3.44 *(1.25-9.54)*		
	50-59	2.47 *(0.87-6.97)*		
	60+	2.65 *(0.91-7.69)*		
**Years Practicing, years**	<2*	-	455	0.07
	2-4	*2.51* *(0.97-6.52)*		
	5-9	2.39 *(0.97-5.89)*		
	10-14	2.59 *(1.05-6.41)*		
	>15	1.64 *(0.70-3.83)*		
**Specialty**	Emergency Medicine	1.63 *(1.17-2.28)*	455	<0.01
	No operation room anaesthesia (NORA)	*1.40* *(1.01-1.95)*	455	0.04
	Neuro-anaesthesia	*1.20* *(0.86-1.68)*	455	0.28
	Cardio-, vascular-, thoracic anaesthesiology	1.17 *(0.84-1.63)*	455	0.36
	Ambulatory anaesthesia	1.15 *(0.41-3.22)*	455	0.80
	General anaesthesiology	*1.29* *(0.85-1.97)*	455	0.24
	Paediatric/neonatal anaesthesiology	1.01 *(0.71-1.41)*	455	0.98
	Obstetric anaesthesiology	*0.99* *(0.71-1.38)*	455	0.95
**Perform Echo**	TOE & TTE	*2.02* *(1.02-4.05)*	455	<0.01
	TTE	2.25 *(1.37-3.71)*		
	TOE	1.00 *(0.67-1.49)*		
	None*	-		
**MRI in Institute**	Yes*	-	454	<0.01
	No	*0.56* *(0.30-1.06)*		
	Not sure	*0.22* *(0.09-0.51)*		
**CMR in Institute**	Yes*	-	404	<0.01
	No	*0.31* *(0.18-0.53)*		
	Not sure	*0.14* *(0.08-0.23)*		
**Number of Operating Rooms, n**	<5*	-	455	0.02

		Statistical Model		
**Ordinal Regression**	**Responses**	**OR (95%-CI)**	**#Obs**	**p-value**
	5-10	*1.48* *(0.78-2.79)*		
	11-19	*1.43* *(0.72-2.86)*		
	20-29	*1.93* *(1.02-3.54)*		
	>30	*2.57* *(1.37-4.82)*		
**Institute**	Private hospital	*1.21* *(0.70-2.11)*	455	0.50
	Tertiary (non-University)	*1.38* *(0.94-2.02)*	455	0.09
	Primary care hospital	*0.78* *(0.42-1.47)*	455	0.46
	Ambulatory anaesthesia provider	*1.15* *(0.41-3.22)*	455	0.80
	University hospital	*0.87* *(0.62-1.21)*	455	0.40
	Secondary care hospital	*0.48* *(0.30-0.76)*	455	0.02
**Region**†	Europe*	-	365	0.02
	Americas	*0.49* *(0.28-0.84)*		
	Eastern Mediterranean	*0.45* *(0.16-1.32)*		

From the statistical model, the adjusted odds ratio (OR) along with 95% confidence intervals and number of observations remaining in the final number (#Obs) are reported. A higher OR is associated with answering Yes to the incorporation of CMR findings.
*Refers to reference category in ordinal regression.
†African, West Pacific and South East Asia are not included in the ordinal logistic regression due to <5 responses.

Supplemental Table 5: Access and Understanding of Clinical CMR Reports

		Frequency (%)			Statistical Model		
**Question**	**Response category**	**All (n=455)**	**CVA (n=255)**	**Non-CVA (n=200)**	**OR (95%-CI)**	**#Obs**	**p-value**
**"I do not know enough about CMR to include it in my clinical practice"**	Strongly agree	68 (15%)	34 (13%)	34 (17%)	0.74 *(0.53-1.04)*	431	0.09
	Agree	127 (28%)	71 (28%)	56 (28%)			
	Neither agree nor disagree	103 (23%)	55 (22%)	48 (24%)			
	Disagree	94 (21%)	58 (23%)	36 (18%)			
	Strongly disagree	34 (8%)	24 (9%)	10 (5%)			
	N/A / Unknown	5 (1%)	2 (1%)	3 (2%)			
	Not answered	24 (5%)	11 (4%)	13 (7%)			
**"I understand the standard reporting on clinical CMR reports well"**	Strongly agree	28 (6%)	18 (7%)	10 (5%)	1.50 *(1.06-2.13)*	428	0.02
	Agree	166 (37%)	104 (41%)	62 (31%)			
	Neither agree nor disagree	120 (26%)	61 (24%)	59 (30%)			
	Disagree	70 (15%)	42 (17%)	28 (14%)			
	Strongly disagree	26 (6%)	12 (5%)	14 (7%)			
	N/A / Unknown	18 (4%)	6 (2%)	12 (6%)			
	Not answered	27 (6%)	12 (5%)	15 (8%)			
**"I do not have access to CMR data in my patients' files"**	Strongly agree	38 (8%)	18 (7%)	20 (10%)	0.84 *(0.60-1.18)*	429	0.32
	Agree	58 (13%)	32 (13%)	26 (13%)			
	Neither agree nor disagree	37 (8%)	20 (8%)	17 (9%)			
	Disagree	119 (26%)	68 (27%)	51 (26%)			
	Strongly disagree	153 (34%)	94 (37%)	59 (30%)			
	N/A / Unknown	24 (5%)	11 (4%)	13 (7%)			
	Not answered	26 (6%)	12 (5%)	14 (7%)			

Frequencies (percentages) are reported. From the statistical model, the adjusted odds ratio (OR) along with 95% confidence intervals and number of observations remaining in the final number (#Obs) are reported. An odds ratio greater than 1 indicates increased use by CVA. CVA: anaesthesiologists who indicated cardio-, vascular-, or thoracic anaesthesiology as an area of clinical expertise.

Supplemental Table 6: Opinions on CMR Literature and Usefulness

		Frequency (%)			Statistical Model		
**Question**	**Responses**	**All (n=455)**	**CVA (n=255)**	**Non-CVA (n=200)**	**OR (95%-CI)**	**#Obs**	**p-value**
**There is not enough literature linking CMR and anaesthesia outcomes**	Strongly agree	26 (6%)	17 (7%)	9 (5%)	1.21 *(0.86-1.71)*	430	0.28
	Agree	104 (23%)	58 (23%)	46 (23%)			
	Neither agree nor disagree	85 (19%)	51 (20%)	34 (17%)			
	Disagree	36 (8%)	24 (9%)	12 (6%)			
	Strongly disagree	4 (1%)	2 (1%)	2 (1%)			
	N/a, unknown	175 (39%)	92 (36%)	83 (42%)			
	Not answered	25 (6%)	11 (4%)	14 (7%)			
**CMR is not a useful tool for anaesthesiologists**	Strongly agree	9 (2%)	5 (2%)	4 (2%)	0.99 *(0.70-1.39)*	429	0.94
	Agree	32 (7%)	18 (7%)	14 (7%)			
	Neither agree nor disagree	79 (17%)	39 (15%)	40 (20%)			
	Disagree	169 (37%)	103 (40%)	66 (33%)			
	Strongly disagree	67 (15%)	44 (17%)	23 (12%)			
	N/a, unknown	73 (16%)	34 (13%)	39 (20%)			
	Not answered	26 (6%)	14 (6%)	12 (6%)			
**I think CMR holds potential for peri-operative risk evaluation**	Strongly agree	66 (15%)	36 (14%)	30 (15%)	1.24 *(0.85-1.79)*	429	0.27
	Agree	251 (55%)	150 (59%)	101 (51%)			
	Neither agree nor disagree	70 (15%)	37 (15%)	33 (17%)			
	Disagree	16 (4%)	9 (4%)	7 (4%)			
	Strongly disagree	3 (1%)	1 (0.4%)	2 (1%)			
	N/a, unknown	23 (5%)	9 (4%)	14 (7%)			
	Not answered	26 (6%)	13 (5%)	13 (7%)			
**I would like to include CMR more into my clinical practice in the future**	Strongly agree	47 (10%)	28 (11%)	19 (10%)	1.06 *(0.75-1.51)*	431	0.74
	Agree	186 (41%)	103 (40%)	83 (42%)			
	Neither agree nor disagree	143 (31%)	87 (34%)	56 (28%)			
	Disagree	23 (5%)	11 (4%)	12 (6%)			
	Strongly disagree	5 (1%)	2 (1%)	3 (2%)			
	N/a, unknown	27 (6%)	13 (5%)	14 (7%)			
	Not answered	24 (5%)	11 (4%)	13 (7%)			

Frequencies (percentages) are reported. CVA: anaesthesiologists who indicated cardio-, vascular-, or thoracic anaesthesiology as an area of clinical expertise. From the statistical model, the adjusted odds ratio (OR) along with 95% confidence intervals and number of observations remaining in the final number (#Obs) are reported. An odds ratio greater than 1 indicates increased use by CVA.

Supplemental Table 7: Use of Individual CMR Parameters

		Frequency (%)			Statistical Model		
**For the most common CMR parameters which statement do you as an anaesthesiologist most agree with?**	**Responses**	**All (n=455)**	**CVA (n=255)**	**Non-CVA (n=200)**	**OR (95 %-CI)**	**#Obs**	**p-value**
**Left ventricular function and anatomy**	I understand this marker well and I often use it clinically when data is available	264 (58%)	158 (62%)	106 (53%)	1.30 *(0.85 - 1.99)*	384	0.23
	I understand this marker well, but I rarely or only sometimes use it clinically	62 (14%)	36 (14%)	26 (13%)			
	I understand this marker well, but I do not use it clinically	23 (5%)	17 (7%)	6 (3%)			
	I know a little bit about this marker, but I do not understand what it is	26 (6%)	11 (4%)	15 (8%)			
	I have never heard of this marker	9 (2%)	2 (<1%)	7 (4%)			
	None of the previous answers	9 (2%)	5 (2%)	4 (2%)			
	Not Answered	62 (14%)	26 (10%)	36 (18%)			
**Right ventricular function and anatomy**	I understand this marker well and I often use it clinically when data is available	225 (50%)	142 (56%)	83 (42%)	1.65 *(1.11 - 2.47)*	378	0.02
	I understand this marker well, but I rarely or only sometimes use it clinically	84 (19%)	47 (18%)	37 (19%)			
	I understand this marker well, but I do not use it clinically	28 (6%)	17 (7%)	11 (6%)			
	I know a little bit about this marker, but I do not understand what it is	32 (7%)	14 (6%)	18 (9%)			
	I have never heard of this marker	9 (2%)	2 (<1%)	7 (4%)			

		Frequency (%)			Statistical Model		
**For the most common CMR parameters which statement do you as an anaesthesiologist most agree with?**	**Responses**	**All (n=455)**	**CVA (n=255)**	**Non-CVA (n=200)**	**OR (95 %-CI)**	**#Obs**	**p-value**
	None of the previous answers	10 (2%)	6 (2%)	4 (2%)			
	Not Answered	67 (15%)	27 (11%)	40 (20%)			
**Atrial function and anatomy**	I understand this marker well and I often use it clinically when data is available	140 (31%)	85 (33%)	55 (28%)	1.24 *(0.86 - 1.79)*	380	0.25
	I understand this marker well, but I rarely or only sometimes use it clinically	119 (26%)	74 (29%)	45 (23%)			
	I understand this marker well, but I do not use it clinically	46 (10%)	25 (10%)	21 (11%)			
	I know a little bit about this marker but I do not understand what it is	60 (13%)	33 (13%)	27 (14%)			
	I have never heard of this marker	15 (3%)	7 (3%)	8 (4%)			
	None of the previous answers	12 (3%)	5 (2%)	7 (4%)			
	Not Answered	63 (14%)	26 (10%)	37 (19%)			
**Aortic function and anatomy**	I understand this marker well and I often use it clinically when data is available	170 (37%)	105 (41%)	65 (33%)	1.34 *(0.92 - 1.95)*	376	0.12
	I understand this marker well, but I rarely or only sometimes use it clinically	90 (20%)	56 (22%)	34 (17%)			
	I understand this marker well, but I do not use it clinically	31 (7%)	17 (7%)	14 (7%)			
	I know a little bit about this marker, but I do not understand what it is	55 (12%)	30 (12%)	25 (13%)			

		Frequency (%)			Statistical Model		
**For the most common CMR parameters which statement do you as an anaesthesiologist most agree with?**	**Responses**	**All (n=455)**	**CVA (n=255)**	**Non-CVA (n=200)**	**OR (95 %-CI)**	**#Obs**	**p-value**
	I have never heard of this marker	30 (7%)	14 (6%)	16 (8%)			
	None of the previous answers	14 (3%)	6 (2%)	8 (4%)			
	Not Answered	65 (14%)	27 (11%)	38 (19%)			
**Valvular function and anatomy**	I understand this marker well and I often use it clinically when data is available	239 (53%)	140 (55%)	99 (50%)	1.05 *(0.70 - 1.59)*	377	0.80
	I understand this marker well, but I rarely or only sometimes use it clinically	68 (15%)	40 (16%)	28 (14%)			
	I understand this marker well, but I do not use it clinically	20 (4%)	13 (5%)	7 (4%)			
	I know a little bit about this marker but I do not understand what it is	35 (8%)	20 (8%)	15 (8%)			
	I have never heard of this marker	15 (3%)	7 (3%)	8 (4%)			
	None of the previous answers	11 (2%)	5 (2%)	6 (3%)			
	Not Answered	67 (15%)	30 (12%)	37 (19%)			
**Inducible myocardial ischaemia (ie. myocardial perfusion, stress CMR)**	I understand this marker well and I often use it clinically when data is available	222 (49%)	136 (53%)	86 (43%)	1.47 *(0.98 - 2.19)*	373	0.06
	I understand this marker well, but I rarely or only sometimes use it clinically	62 (14%)	38 (15%)	24 (12%)			
	I understand this marker well, but I do not use it clinically	23 (5%)	13 (5%)	10 (5%)			
	I know a little bit about this marker but I do not	50 (11%)	27 (11%)	23 (12%)			

		Frequency (%)			Statistical Model		
**For the most common CMR parameters which statement do you as an anaesthesiologist most agree with?**	**Responses**	**All (n=455)**	**CVA (n=255)**	**Non-CVA (n=200)**	**OR (95 %-CI)**	**#Obs**	**p-value**
	understand what it is						
	I have never heard of this marker	16 (4%)	4 (2%)	12 (6%)			
	None of the previous answers	17 (4%)	9 (4%)	8 (4%)			
	Not Answered	65 (14%)	28 (11%)	37 (19%)			
**Myocardial scarring (ie. late gadolinium enhancement, delayed enhancement, tissue viability)**	I understand this marker well and I often use it clinically when data is available	132 (29%)	86 (34%)	46 (23%)	1.66 *(1.14 - 2.41)*	373	<0.01
	I understand this marker well, but I rarely or only sometimes use it clinically	88 (19%)	57 (22%)	31 (16%)			
	I understand this marker well, but I do not use it clinically	37 (8%)	17 (7%)	20 (10%)			
	I know a little bit about this marker but I do not understand what it is	82 (18%)	44 (17%)	38 (19%)			
	I have never heard of this marker	34 (8%)	15 (6%)	19 (10%)			
	None of the previous answers	16 (4%)	7 (3%)	9 (5%)			
	Not Answered	66 (15%)	29 (11%)	37 (19%)			
**Myocardial diffuse fibrosis / extracellular volume ie. (T1, ECV)**	I understand this marker well and I often use it clinically when data is available	54 (12%)	40 (16%)	14 (7%)	2.33 *(1.60 - 3.40)*	372	<0.01
	I understand this marker well, but I rarely or only sometimes use it clinically	73 (16%)	51 (20%)	22 (11%)			
	I understand this marker well, but I do not use it clinically	36 (8%)	21 (8%)	15 (8%)			
	I know a little bit about this marker	116 (26%)	65 (26%)	51 (26%)			

		Frequency (%)			Statistical Model		
**For the most common CMR parameters which statement do you as an anaesthesiologist most agree with?**	**Responses**	**All (n=455)**	**CVA (n=255)**	**Non-CVA (n=200)**	**OR (95 %-CI)**	**#Obs**	**p-value**
	but I do not understand what it is						
	I have never heard of this marker	93 (20%)	39 (15%)	54 (27%)			
	None of the previous answers	18 (4%)	10 (4%)	8 (4%)			
	Not Answered	65 (14%)	29 (11%)	36 (18%)			
**Myocardial edema (ie. T2, T2STIR)**	I understand this marker well and I often use it clinically when data is available	44 (10%)	35 (14%)	9 (5%)	2.42 *(1.66 - 3.53)*	375	<0.01
	I understand this marker well, but I rarely or only sometimes use it clinically	68 (15%)	51 (20%)	17 (9%)			
	I understand this marker well, but I do not use it clinically	49 (11%)	25 (10%)	24 (12%)			
	I know a little bit about this marker, but I do not understand what it is	104 (23%)	58 (23%)	46 (23%)			
	I have never heard of this marker	110 (24%)	49 (19%)	61 (31%)			
	None of the previous answers	16 (4%)	9 (4%)	7 (4%)			
	Not Answered	64 (14%)	28 (11%)	36 (18%)			

Frequencies (percentages) are reported. CVA: anaesthesiologists who indicated cardio-, vascular-, or thoracic anaesthesiology as an area of clinical expertise. From the statistical model, the adjusted odds ratio (OR) along with 95% confidence intervals and number of observations remaining in the final number (#Obs) are reported. The answer "none of the previous answers" was not included into the ordinal regression. An odds ratio greater than 1 indicates increased use by CVA.

Supplemental Table 8. Assessment of Multicollinearity

Variable	**Incorporation of CMR findings**	Age	Years practicing	Cardio-, vascular-, thoracic anaesthesiology	Not individually perform echocardiographic imaging	Hospital having MRI	Hospital performing CMR	Number of operating rooms
**Incorporation of CMR findings**		0.21 (<0.01)	0.20 (<0.01)	-0.08 (0.07)	-0.02 (0.73)	0.18 (<0.01)	0.34 (<0.01)	0.06 (0.15)
Age			0.85 (<0.01)	-0.05 (0.23)	-0.07 (0.13)	0.09 (0.05)	0.08 (0.12)	-0.00 (0.94)
Years practicing				-0.04 (0.42)	-0.11 (0.01)	0.05 (0.25)	-0.00 (0.96)	0.04 (0.43)
Cardio-, vascular-, thoracic anaesthesiology					-0.16 (<0.01)	-0.06 (0.17)	-0.09 (0.06)	0.13 (0.01)
Not individually perform echocardiographic imaging						0.23 (<0.01)	0.20 (<0.01)	-0.09 (0.05)
Hospital having MRI							0.61 (<0.01)	-0.02 (0.62)
Hospital performing CMR								-0.10 (0.04)
Number of operating rooms								

**Institute Type**								
University hospital	0.02 (0.69)	-0.19 (<0.01)	-0.17 (<0.01)	0.22 (<0.01)	0.03 (0.57)	-0.01 (0.88)	-0.09 (0.06)	0.44 (<0.01)
Tertiary care hospital	-0.04 (0.37)	-0.08 (0.07)	-0.10 (0.03)	0.22 (<0.01)	0.05 (0.24)	0.11 (0.01)	0.12 (0.01)	-0.04 (0.38)
Secondary care hospital	0.18 (<0.01)	0.10 (0.02)	0.08 (0.07)	-0.06 (0.22)	0.23 (<0.01)	0.13 (<0.01)	0.27 (<0.01)	-0.25 (<0.01)
Primary care hospital	0.13 (<0.01)	0.15 (<0.01)	0.09 (0.04)	-0.02 (0.63)	0.17 (<0.01)	0.24 (<0.01)	0.27 (<0.01)	-0.13 (<0.01)
Private hospital	0.04 (0.33)	0.09 (0.05)	0.10 (0.04)	0.12 (0.01)	0.08 (0.06)	0.24 (<0.01)	0.21 (<0.01)	-0.09 (0.04)
Ambulatory anaesthesia provider	0.14 (<0.01)	0.09 (0.04)	0.11 (0.02)	0.09 (0.05)	0.17 (<0.01)	0.30 (<0.01)	0.25 (<0.01)	0.04 (0.40)

Pairwise associations between variables were assessed using Spearman's rank correlation to explore potential collinearity, with coefficients (ρ) and corresponding p-values (in brackets) presented in a correlation matrix. Correlation coefficients were interpreted as weak ($\rho < 0.3$), moderate ($\rho = 0.3$–0.6), and strong ($\rho > 0.6$), with particular attention to values exceeding 0.7–0.8 as suggestive of potentially problematic collinearity.

There is some expected multicollinearity such as age of the anaesthetist and years practicing, having on-site CMR (cardiovascular magnetic resonance) services in institutes with an MRI (magnetic resonance imaging) machine. Nevertheless, few variables show a high collinearity, suggesting that multicollinearity is unlikely to be of significant concern. Moreover, these collinear variables were not assessed together in the same model.
